# A growth-year classification model for cultivated ginseng based on feature–decision-level fusion of leaf hyperspectral and RGB image features

**DOI:** 10.3389/fpls.2026.1889841

**Published:** 2026-09-21

**Authors:** Yifeng Zhu, Danyang Yu, Shaozhong Song, Xu Yang, Xiaofeng An, Yanqiu Li

**Affiliations:** 1 School of Electronic Information Engineering, Changchun University of Science and Technology, Changchun, China; 2 School of Data Science and Artificial Intelligence, Jilin Engineering Normal University, Changchun, China; 3 Education Quality Monitoring Center, Jilin Engineering Normal University, Changchun, China

**Keywords:** decision-level fusion, feature-level fusion, growth year of cultivated ginseng, hyperspectral, ResNet18, RGB images, SVM

## Abstract

The growth year of cultivated ginseng is an important factor influencing its quality and price. This study established a dataset comprising leaves from 1- to 5-year-old cultivated ginseng plants. With the aim of distinguishing the growth years of cultivated ginseng plants, a machine-learning and deep-learning dual-branch fusion non-destructive identification model was developed. In the machine-learning branch, 19 spectral features were derived using the leaf hyperspectral data and an SVM identification model was built. The accuracy of this model was 90.89%. The accuracy of the SVM model was improved to 94.58% through the extraction of six RGB image-derived phenotypic features and the use of feature fusion with spectral data. In order to learn deeper features in the images, the ResNet18 model was created in the deep-learning branch based on RGB images. Finally, the predictions of the feature-fusion-based SVM model and the ResNet18 model were combined with the help of decision weighted voting fusion, which gave the overall accuracy of 96.80%. It is an increase of 5.91 percentage points when compared to the spectral-feature-based SVM model and an increase of 2.22 percentage points when compared to the feature-fusion-based SVM model, and the overall relative improvement rate is 6.50%. The findings show that the fusion of leaf hyperspectral features and RGB image-derived phenotypic features, and the decision fusion of SVM and ResNet18, can be used to progressively improve the accuracy of identifying the growth year of cultivated ginseng. It offers a new approach to quality evaluation of cultivated ginseng and identifying its growth year.

## Introduction

Ginseng (Panax ginseng) is one of the most widely used traditional Chinese medicinal herbs. Ginseng contains active compounds such as saponins and polysaccharides, which help in enhancing the immune system and slow down the aging process (Choi, 2008). Wild ginseng has become scarce in recent years, and the market supply depends much on cultivated ginseng (Baeg and So, 2013). Being a cultivated variety of ginseng, the content of active compounds like ginsenosides in cultivated ginseng changes depending on its growth year (Shi et al., 2007), which in turn influences its medicinal value. Consequently, growth year has become an important criterion for quality assessment of cultivated ginseng.

The common methods used in identifying the growth year of cultivated ginseng mainly depend on its morphology, weight, and human experience. This method is highly subjective, and the results are unstable. Recently, spectroscopic techniques have been used in the task of growth-year identification of ginseng. Pan et al. were able to distinguish among ginseng powder tablet samples with growth years of 4, 5, and 6 years by integrating terahertz spectroscopy with support vector machine (Pan et al., 2021). Zhao et al. took tablets made from ginseng powder with growth years of 2 and 4 years as samples and used laser-induced breakdown spectroscopy (LIBS) and hyperspectral imaging (HSI) to perform growth-year identification of ginseng (Zhao et al., 2021). Limin Zhao et al. classified ginseng root samples with growth years of 1–7 years using hyperspectral data in the 400–2,500 nm range and the random forest model (Zhao et al., 2022). Powder compression is a destructive sampling method; it is difficult to perform non-destructive growth-year identification of growing ginseng with ginseng roots as samples, and this method is difficult to implement quickly and on a large scale in actual production. Consequently, there is an urgency to have a rapid, non-destructive, and accurate method for growth-year identification of cultivated ginseng in order to improve quality control standards.

It is simple to gather leaves, and they have physiochemical information like pigments, water content, and dry matter that affect the spectral features of leaves (Jacquemoud et al., 1996). Hyperspectral imaging technology was used by Debnath et al. in the wavelength range of 380–1000 nm to identify the age of grape leaves and it has been observed that spectral responses are associated with variations in chlorophyll, nitrogen, and protein content during the leaf senescence process (Debnath et al., 2019). Color, morphology, and texture are external characteristics of leaves that can be measured by RGB images (Li et al., 2014). The use of color and texture features in RGB images of citrus leaves has been employed in previous studies to identify plant diseases (Pydipati et al., 2006). Thus, using cultivated ginseng leaves as samples holds promise for providing a rapid, non-destructive method for growth-year identification of growing cultivated ginseng plants.

Over the last few years, multimodal data fusion has become a common practice in non-destructive detection and classification in the field of plant research. Various imaging and sensing modalities are able to provide complementary data, including structural, biochemical, and phenotypic features, and the combination of various modalities is capable of addressing the limitations of single-modal analysis. Yang et al. transformed one-dimensional hyperspectral data into two-dimensional spectral reflectance curve images and combined them with RGB images to achieve data-level fusion for sorghum seed variety classification (Yang et al., 2025). Jia et al. combined UAV-based RGB data with hyperspectral-based information and compared early fusion at the input level with middle fusion after dual-branch feature extraction. The early-fusion model increased the mAP from 83.21% for the RGB-only model to 86.65% (Jia et al., 2026). Sun et al. also performed feature-level fusion of RGB color indices and multispectral vegetation indices, which enhanced the prediction-set R² of the SVR model from 0.866 to 0.878 (Sun et al., 2025).

Multimodal data fusion has also gained popularity in ginseng studies as an approach to geographical origin identification, seed classification, and quality evaluation. Jiang et al. reviewed the use of different spectroscopic and imaging methods to authenticate and assess the quality of ginseng and emphasized that the combination of multimodal data fusion with machine learning or deep learning to perform classification and regression modeling could address the information constraints of single-modal methods (Jiang et al., 2026). Xue et al. combined the morphological characteristics of the RGB images with hyperspectral information by using recursive feature elimination (RFE) and competitive adaptive reweighted sampling (CARS) to choose the important features. They later created a random forest model optimized using the improved red-billed blue magpie optimization algorithm (ORBMO-RF) to allow nondestructive classification of various types of ginseng seeds (Xue et al., 2026). Zhang et al. developed a multimodal data-fusion framework incorporating laser-induced breakdown spectroscopy (LIBS) and hyperspectral imaging (HSI). High-dimensional fused features were reduced in dimensionality and denoised with the use of principal component analysis (PCA), after which machine-learning algorithms such as support vector machine (SVM), random forest (RF), and k-nearest neighbors (KNN) were used to trace the geographical origin of ginseng under small-sample conditions (Zhang et al., 2026).

This study used 1- to 5-growth-year cultivated ginseng leaves as the research subject and proposed a method for the rapid, non-destructive growth-year identification of cultivated ginseng through multimodal fusion of spectral features and RGB image-derived phenotypic features. The experimental procedure is shown in **Figure 1**. The major contributions of this paper are: (1) We selected 19 characteristic wavelengths in the hyperspectral data that are closely related to the growth year and we extracted six RGB image-derived phenotypic features from the RGB images that reflect the growth year. (2) The characteristic wavelengths and image-derived phenotypic features are fused at the feature level to build a multimodal fusion feature set. (3) Using a probability-based soft voting strategy weighted by F1-score across different categories, we propose a decision-level fusion model combining machine-learning and deep-learning in order to improve the accuracy of growth-year identification.

**Figure 1:**
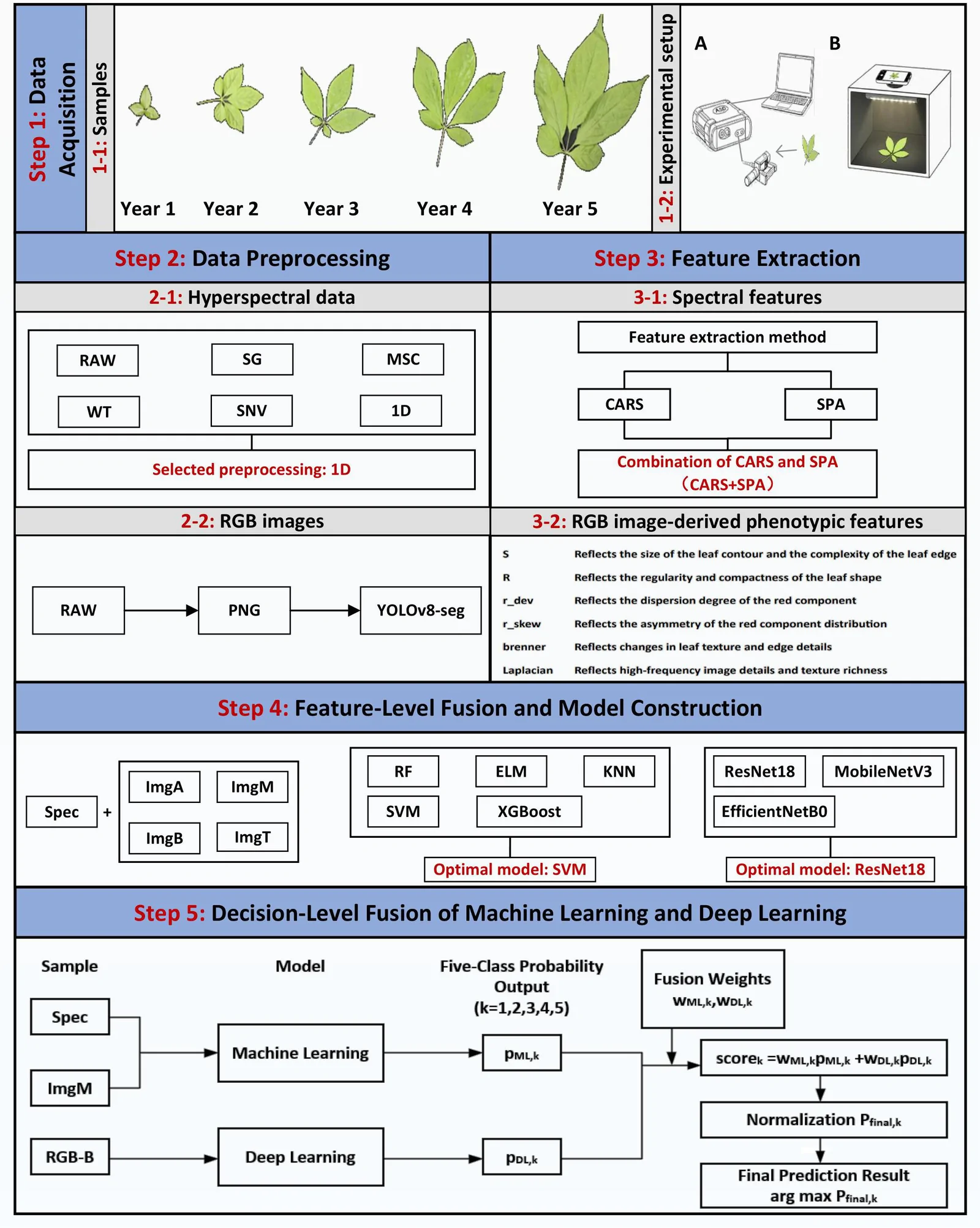
Technical route of the proposed method.

## Materials and methods

### Test sample

The experiment was conducted at the Jiutai Research Base of the Institute of Special Animal and Plant Sciences, Chinese Academy of Agricultural Sciences, located in Jiutai District, Changchun, Jilin Province, China (125.5°E, 44.9°N). In order to reduce the effects of direct sunlight and rain, the cultivated ginseng plants were covered with shade netting (**Figure 2**). The data were collected on July 2, 2025. Leaves showing no obvious signs of pests or diseases were collected from 1-, 2-, 3-, 4-, and 5-year-old plants, with each leaf taken from a different plant (**Table 1**). A total of 406 leaf samples were collected, including 72 from one-year-old plants, 82 from two-year-old plants, 81 from three-year-old plants, 82 from four-year-old plants, and 89 from five-year-old plants.

**Figure 2:**
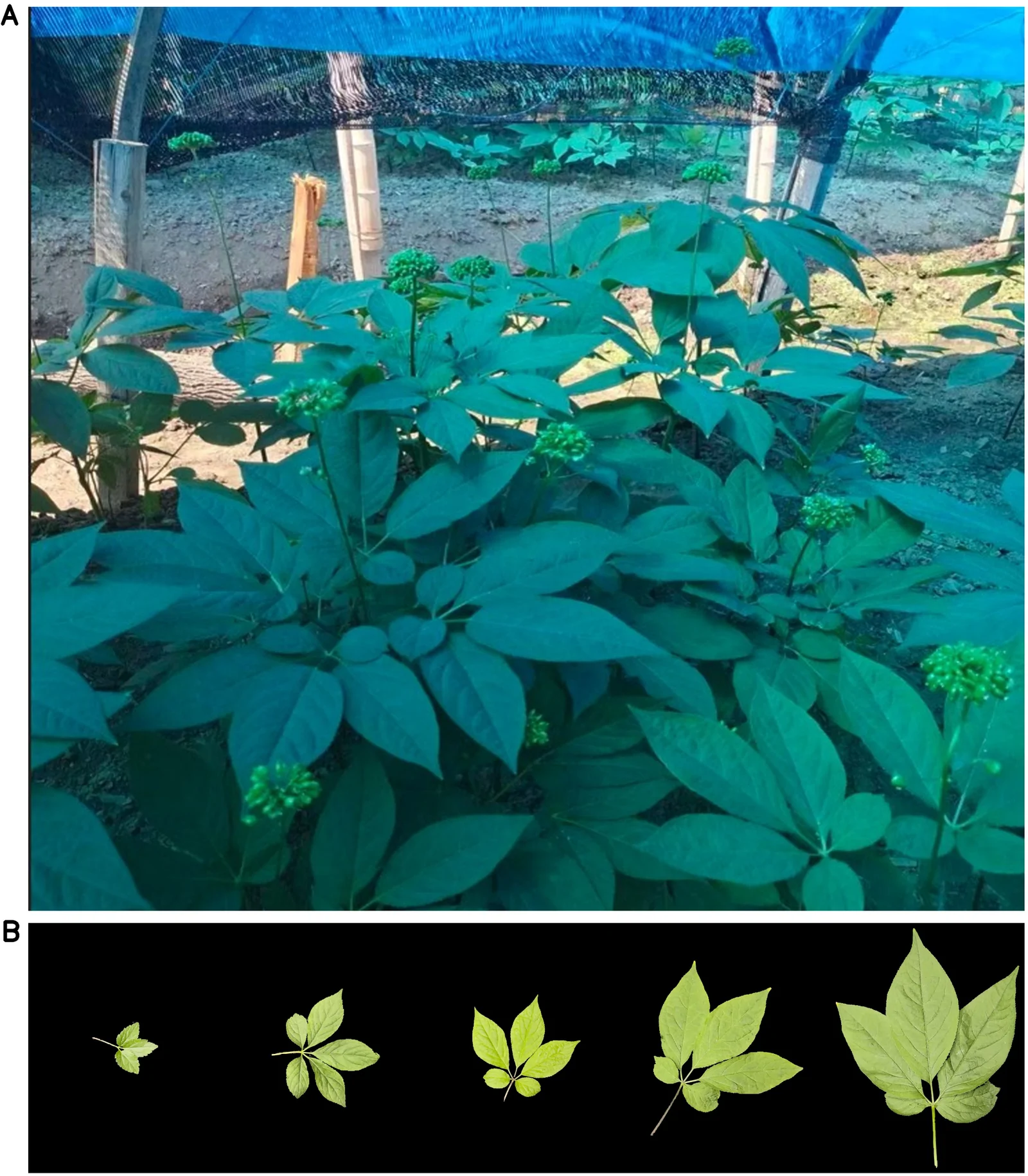
Field cultivation environment and representative leaves of cultivated ginseng. **(A)** Field cultivation environment of cultivated ginseng. **(B)** Representative leaves from 1- to 5-year-old cultivated ginseng.

**Table 1:** Comparison of accuracy (%) for five classification algorithms under different preprocessing methods.

Models	RAW	SG	WT	MSC	SNV	1D
RF	64.29	61.82	63.05	64.53	64.78	87.44
XGBoost	67.98	66.26	67.98	67.49	67.49	91.38
ELM	48.03	47.29	47.54	56.65	52.71	84.24
KNN	54.19	54.19	54.19	57.39	59.85	78.08
SVM	59.36	59.36	59.36	64.29	65.27	91.38

Bold values indicate the highest classification accuracy achieved by each model under different preprocessing methods.

### Hyperspectral data

#### Hyperspectral data acquisition

Hyperspectral data were collected using an ASD FieldSpec4 field spectrometer (Analytical Spectral Devices, USA; 350–2500 nm wavelength range). The probe, when used in conjunction with a leaf clamp, effectively minimizes interference from the external environment (**Figure 3A**). When taking measurements, avoid the leaf veins and try to measure the same spot on each leaf. Use a whiteboard for calibration before each measurement, and calculate the average after taking 10 consecutive measurements for each leaf.

**Figure 3:**
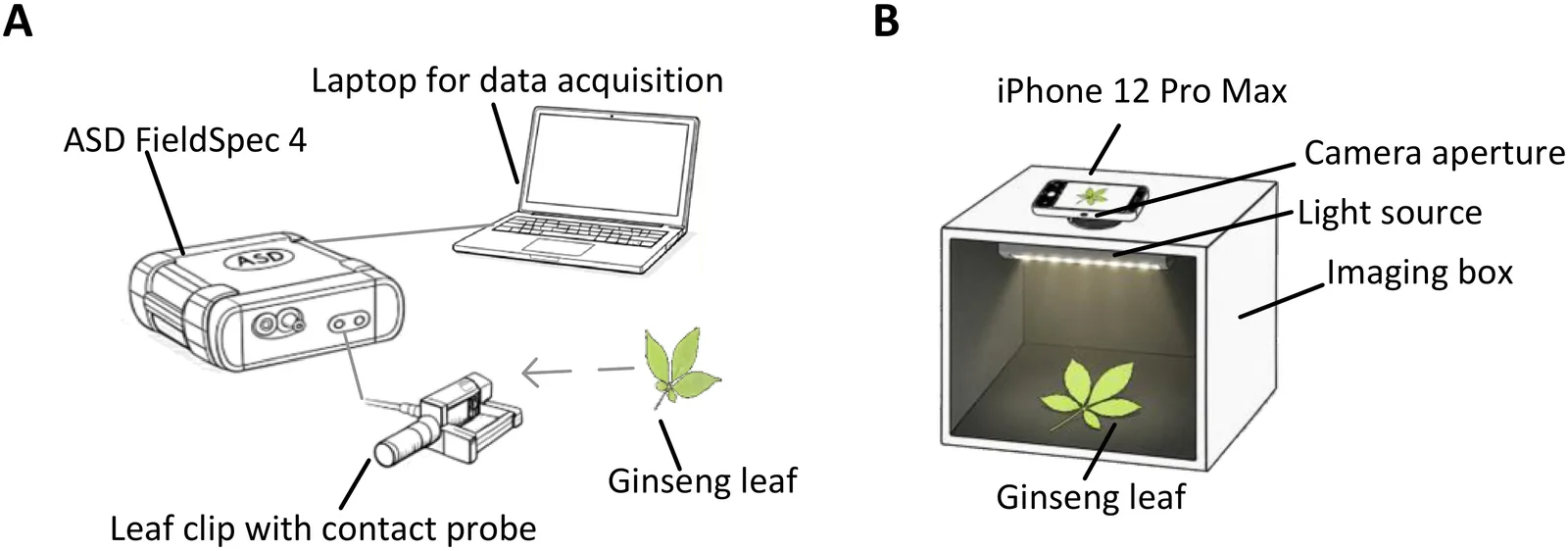
Experimental setup: **(A)** spectral data acquisition; **(B)** image data acquisition.

#### Spectral preprocessing

Use ViewSpec Pro software (Analytical Spectral Devices, Colorado, USA) to export the spectral data of the cultivated ginseng leaves. To minimize the effects of light scattering, baseline drift, and system noise, this study compared five commonly used preprocessing methods. Savitzky–Golay filtering (SG) effectively suppresses high-frequency noise but preserves the overall shape of the spectrum (Liu et al., 2019); Multiplicative Scatter Correction (MSC) is used to correct for the additive and multiplicative effects caused by scattering (Geladi et al., 1985); Wavelet Transform (WT) removes noise from spectral signals by multiscale decomposition and reconstruction of the signal (Alsberg et al., 1997); Standard Normal Variate (SNV) is used to correct for multiplicative noise due to scattering effects and particle size variation (Van Haute et al., 2023);First Derivative (1D): This approach can be used to reduce the effects of background trends and emphasize the detailed characteristics of spectral variation with wavelength by using the differential operation (Zhou et al., 2024).

Five candidate spectral preprocessing methods were independently evaluated using the same five-fold cross-validation procedure, and the preprocessing strategy used in subsequent experiments was selected based on a comparison of their cross-validation performance.

#### Characteristic wavelength selection

There is multicollinearity among the bands in spectral data; this study uses a characteristic wavelength selection algorithm in order to reduce feature redundancy by reducing dimensionality. Competitive Adaptive Reweighted Sampling (CARS) is a combination of Monte Carlo sampling and partial least squares, evaluating band importance based on regression coefficients (Li et al., 2009); The Successive Projections Algorithm (SPA), based on the principle of vector projection, can effectively reduce multicollinearity among spectral bands (Araújo et al., 2001).

CARS and SPA were first applied to identify characteristic wavelengths and construct a fixed spectral feature set. The classification performance of the resulting feature set was subsequently evaluated using five-fold cross-validation with machine-learning classifiers.

### Image data

#### Image acquisition and preprocessing

Images of the leaves were captured using the rear camera of an iPhone 12 Pro Max (Apple Inc., USA). Put the leaf flat in the center of the black light-absorbing cloth, and place the camera 25 cm above the leaf (**Figure 3B**). Images were taken of the upper surface (A) and the lower surface (B) of every leaf; the data collection was done under identical shooting conditions, and the shooting mode and camera settings were kept the same during the whole process. All captured images are saved in RAW format.

Every image was subjected to a unified preprocessing workflow. The first step was the conversion of RAW-format images to PNG format. Then, manual annotation of leaf regions with polygon contours was performed on an RGB image subset to produce leaf segmentation masks. The manually annotated leaf images were split into training and validation sets, on which the YOLOv8-seg model was fine-tuned using pre-trained weights. Following the training, the optimized YOLOv8-seg model was applied to automatically segment the remaining unlabeled RGB images, create leaf region masks, and eliminate interference from the black background and unrelated regions. The created leaf masks were manually checked to ensure that the process of extracting leaf regions was accurate.

#### Extraction of image-derived phenotypic features

RGB images can be used to reflect differences between the external phenotypes of cultivated ginseng leaves in different growth years; in the present research, a total of six representative morphological, color, and texture features were extracted. The image-derived phenotypic features are perimeter (S); rectangularity (R); variance of the red channel (r_dev); skewness of the red channel (r_skew); Brenner gradient (brenner); and Laplacian variance (Laplacian). The specific meanings are given in **Table 2**.

**Table 2:** RGB image-derived phenotypic features and their meanings.

Feature category	Symbol	Meaning
Morphological	*S*	Reflects the size of the leaf contour and the complexity of the leaf edge.
	*R*	Reflects the regularity and compactness of the leaf shape.
Color	*r*_dev_	Reflects the dispersion degree of the red component.
	*r*_skew_	Reflects the asymmetry of the red component distribution.
Texture	Brenner	Reflects changes in leaf texture and edge details.
	Laplacian	Reflects high-frequency image details and texture richness.

### Feature-level fusion

The objective of this study is to make full use of the information contained in hyperspectral data and RGB images and improve the model’s classification performance by using a feature-level fusion strategy. The hyperspectral data of every leaf corresponds to two RGB images: one of the upper surface of the leaf (A) and one of the lower surface (B). Six phenotypic features were obtained from each of the two sides of the leaf (A and B), resulting in the datasets ImgA and ImgB. In order to fully utilize the features of sides A and B, an averaging of ImgA and ImgB is performed element-by-element to yield the dataset ImgM, and the feature vectors of ImgA and ImgB are fused to yield the dataset ImgT.

This study uses a feature-level fusion strategy, which is the concatenation of the spectral features (Spec) of the same leaf sample with the corresponding four RGB image-derived phenotypic feature datasets to obtain four fused feature datasets (SpecImg A; SpecImg B; SpecImg M; SpecImg T).

### Selection of machine-learning classification models

We have used PyCharm with a Python 3.9.0 environment to perform modeling of spectral feature sets, RGB image-derived phenotypic feature sets, and fused feature sets. The evaluation of the model was done through a five-fold stratified cross-validation. All samples were stratified into five subsets based on their class labels. In every iteration, four subsets were used for training the model and the other subset for testing. It was done five times, making sure that each subset was used once as the test set. Before model training, all features were standardized to reduce the influence of differences in feature scales. The standardization parameters were fitted using only the training subset in each fold and were then applied to the corresponding test subset.Random Forest (RF) increases the prediction accuracy by combining the results of multiple decision trees (Pal, 2005); Extreme Gradient Boosting (XGBoost) sequentially builds multiple CART decision trees within the gradient boosting framework and optimizes the loss function using gradient descent (Chen and Guestrin, 2016); The Extreme Learning Machine (ELM) is an algorithm that uses a single-hidden-layer feedforward neural network to solve for the output weights in one step, which involves randomly initializing the hidden-layer weights and using the generalized inverse matrix (Ding et al., 2015); k-nearest neighbor (KNN): The distance between the sample to be classified and training samples is computed and the K nearest samples are chosen as the nearest neighbors and the class of the sample to be classified is decided by their class distribution (Cover and Hart, 1967); Support Vector Machine (SVM) relies on the idea of minimizing structural risk to obtain the optimal separating hyperplane in a high-dimensional feature space (Cortes and Vapnik, 1995).

All machine-learning models were implemented using their default hyperparameter settings. The SVM classifier used in this study was based on a radial basis function (RBF) kernel with the penalty parameter C = 1.0 and the kernel coefficient γ = scale.

The candidate machine-learning classifiers were independently evaluated using the same five-fold stratified cross-validation procedure, and the best-performing classifier was selected based on a comparison of their cross-validation performance.

### Decision-level fusion of machine-learning and deep-learning models

#### Building deep-learning models

For the deep-learning experiments, RGB images of the upper (A) and lower (B) leaf surfaces were separately used as model inputs for classification. The assessment of deep learning models was carried out through five-fold cross-validation. All samples were divided into five subsets. In every iteration, one subset (20 percent of all samples) was used as an independent test set and the other four subsets were subdivided into training (68 percent) and validation (12 percent) sets for model optimization. The procedure was repeated five times, ensuring that every subset was used as a test set once.

ResNet-18 applies residual connections to mitigate the degradation problem in the training of deep neural networks (He et al., 2016); MobileNetV3 is based on a lightweight network architecture which reduces the number of model parameters but still maintains strong feature extraction capabilities (Howard et al., 2019); EfficientNetB0 is a well-balanced network in terms of network depth, width, and input resolution and is based on a compound scaling strategy, improving the classification efficiency of the model (Tan and Le, 2019).

The ResNet18, MobileNetV3, and EfficientNetB0 models were trained under the same experimental conditions. Each model used ImageNet-pretrained weights and was optimized with the AdamW optimizer, with a starting learning rate of 1 × 10−4 and a weight decay of 1 × 10−4. All input images were resized to 224 × 224 pixels, with a batch size of 32 and a maximum of 40 training epochs. To improve model generalization and reduce overfitting, data augmentation was applied only to the training set, including random horizontal flipping, random rotation, and random affine transformations. Data augmentation was not applied to the validation and test sets.

#### Decision-level fusion strategy

Popular probability-based soft voting techniques usually involve combining the class probabilities obtained by various classifiers by means of equal averaging or fixed model-level weighting (Kittler et al., 1998; Sagi and Rokach, 2018). These global weighting methods tend to assume that the relative contributions of various models are constant in every category, disregarding the variations in classification performance across the individual classes. As opposed to that, the current paper uses a class-specific weighting strategy, and the fusion weights of each growth-year category are calculated individually based on the F1-scores that the machine-learning models and deep-learning models scored on the validation set. Hence, the two model contributions towards the end prediction can be different in the various growth-year categories, and the suggested fusion strategy is able to utilize the complementary classification benefits offered by the two models.

Machine-learning models and deep-learning models were first evaluated separately to classify 1- to 5-year-old samples. After selecting the optimal machine-learning model and deep-learning model, the SVM and ResNet18 models were trained using the same data split, including 68% of the samples for training, 12% for validation, and 20% for testing. The fusion strategy is shown in **Figure 4**.

**Figure 4:**
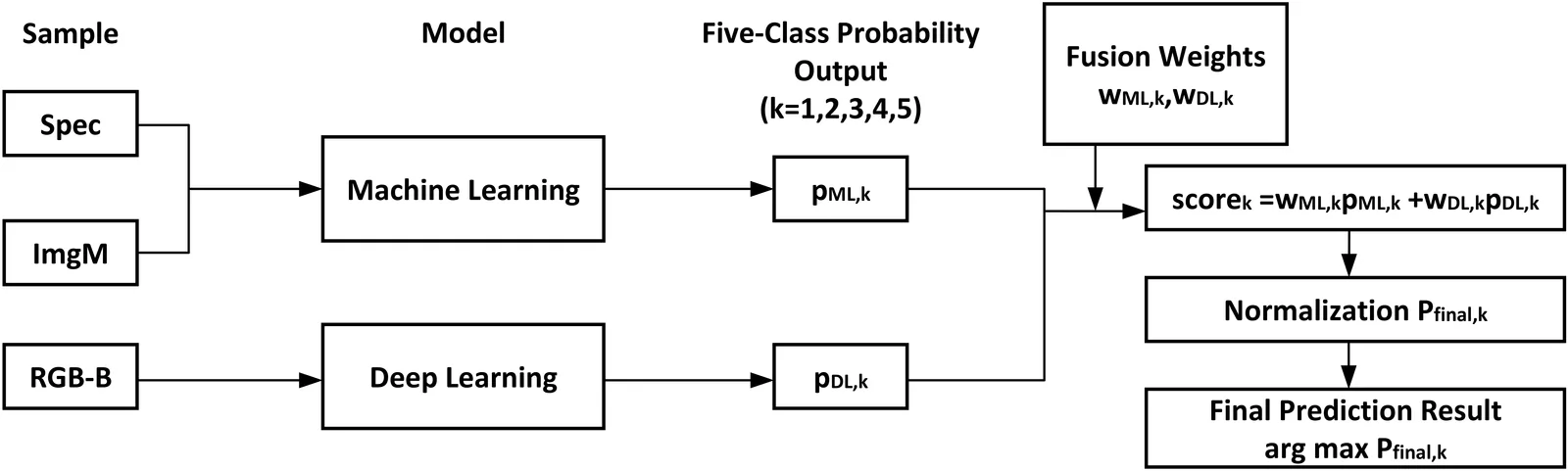
Decision-level fusion strategy based on F1-score-weighted soft voting.

In the five-fold cross-validation process, the fusion weights were estimated separately in each fold. In each fold, the SVM and ResNet18 models were initially trained using the training subset. Then, the validation subset of that fold was used to evaluate the class-specific F1-scores of the two models, and the class-specific fusion weights were computed based on the validation F1-scores. Thereafter, the obtained weights were used to fuse the prediction probabilities of the corresponding test subset. No weights were calculated using the test subset, and it was only used to assess the performance of the final model.

A machine-learning model predicts the probability that a sample belongs to class *k* as *p_ML_*,*_k_*, and a deep-learning model predicts the probability that a sample belongs to class *k* as *p_DL_*,*_k_*, where *k* = 1,2,3,4,5. This study takes into consideration the classification performance of different models across different growth years and chooses the fusion weights according to the F1-scores of each model for the corresponding categories in the validation set, denoted as *w_ML_*,*_k_* and *w_DL_*,*_k_*. The fusion score for the *k*th class is:


$$score_{k}=w_{ML,k}\times p_{ML,k}+w_{DL,k}\times p_{DL,k}$$

Normalize the fusion scores to obtain fused probabilities for the five classes:


$$P_{final,k}=\frac{score_{k}}{\sum_{j=1}^{5}score_{j}}$$

Take the class with the highest fused probability as the final predicted class:


$$\hat{y}=\arg\underset{k\in \left\{1,2,3,4,5\right\}}{\max}P_{final,k}$$

### SHAP-based feature importance analysis

The TreeSHAP algorithm was applied to explain the Random Forest model, wherein SHAP values were calculated with the help of the SHAP TreeExplainer. Mean absolute SHAP values averaged over samples and classes were employed to measure global feature importance, which was later normalized as percentages to indicate the contribution of each feature in relative terms.

SHAP analysis was performed only for model interpretation and was not involved in feature selection or model performance evaluation.

### Model evaluation

The study evaluated model performance using accuracy, precision, recall, F1-score, and a confusion matrix. Accuracy is the ratio of the samples correctly classified by the model to the total number of samples. Precision is the ratio of the samples correctly classified by the model as belonging to a particular class, and Recall is the ratio of the samples in that class that were correctly identified. The F1-score, calculated as the harmonic mean of Precision and Recall, is used to comprehensively evaluate classification performance. A confusion matrix is a tool that can be used to show the relationship between the true labels and the predicted labels for each class.


$$Accuracy=\frac{TP+TN}{TP+FP+TN+FN}$$

$$Precision=\frac{TP}{TP+FP}$$

$$Recall=\frac{TP}{TP+FN}$$

$$F1-score=2\times \frac{Precision\times Recall}{Precision+Recall}$$

## Results

### Spectral preprocessing

As shown in **Figure 5**, the raw spectral curves of the cultivated ginseng leaves were obtained as well as the spectral curves after five preprocessing methods. This spectral range is 350–2500 nm with similar spectral trends between leaf samples from different growth years. After 1D preprocessing, the obtained curve showed the greatest deviation from the original curve, and major peaks were observed at the wavelengths of approximately 521, 710, 1001, 1219, 1493, and 1999 nm, and major troughs at the wavelengths of approximately 568, 953, 1143, 1392, and 1879 nm.

**Figure 5:**
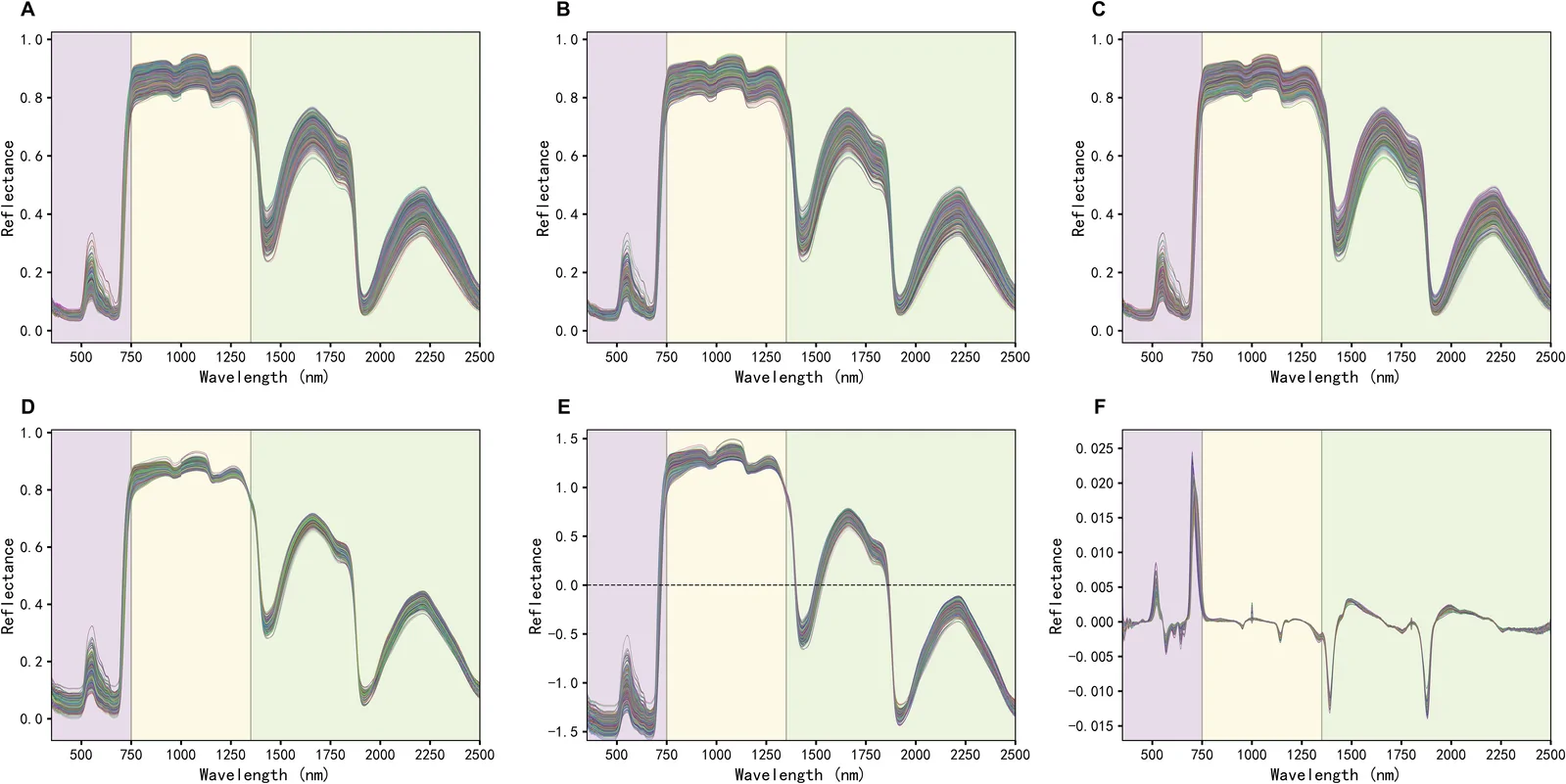
Spectral curves of cultivated ginseng leaves under different preprocessing methods: **(A)** RAW; **(B)** SG; **(C)** WT; **(D)** MSC; **(E)** SNV; **(F)** 1D.

This paper compares the results of five preprocessing methods (**Table 1**). The 1D preprocessing method had the highest accuracy in all models, as 1D is effective at reducing the impact of factors like baseline drift (Savitzky and Golay, 1964). This 1D+SVM model achieved an accuracy of 91.38%, which is an increase of 32.02 percentage points over the RAW+SVM model. Therefore, we selected a 1D method for data preprocessing.

### Spectral feature extraction

#### Comparison of different feature extraction methods

The full-spectrum data (350–2500 nm) has a total of 2,151 spectral bands and is highly redundant. This study compares three methods, namely CARS, SPA, and CARS-SPA, in order to screen for characteristic wavelengths, identify the optimal feature set, and enhance the efficiency of modeling.

**Figure 6** shows the process of selecting characteristic wavelengths using CARS. In the 20th sampling iteration, 130 characteristic wavelengths were selected, and the SVM-based model achieved an F1-score of 92.4% (**Figure 6C**). This feature set represents a good compromise between classification performance and low RMSECV; therefore, it is the optimal choice (**Figure 6**).

**Figure 6:**
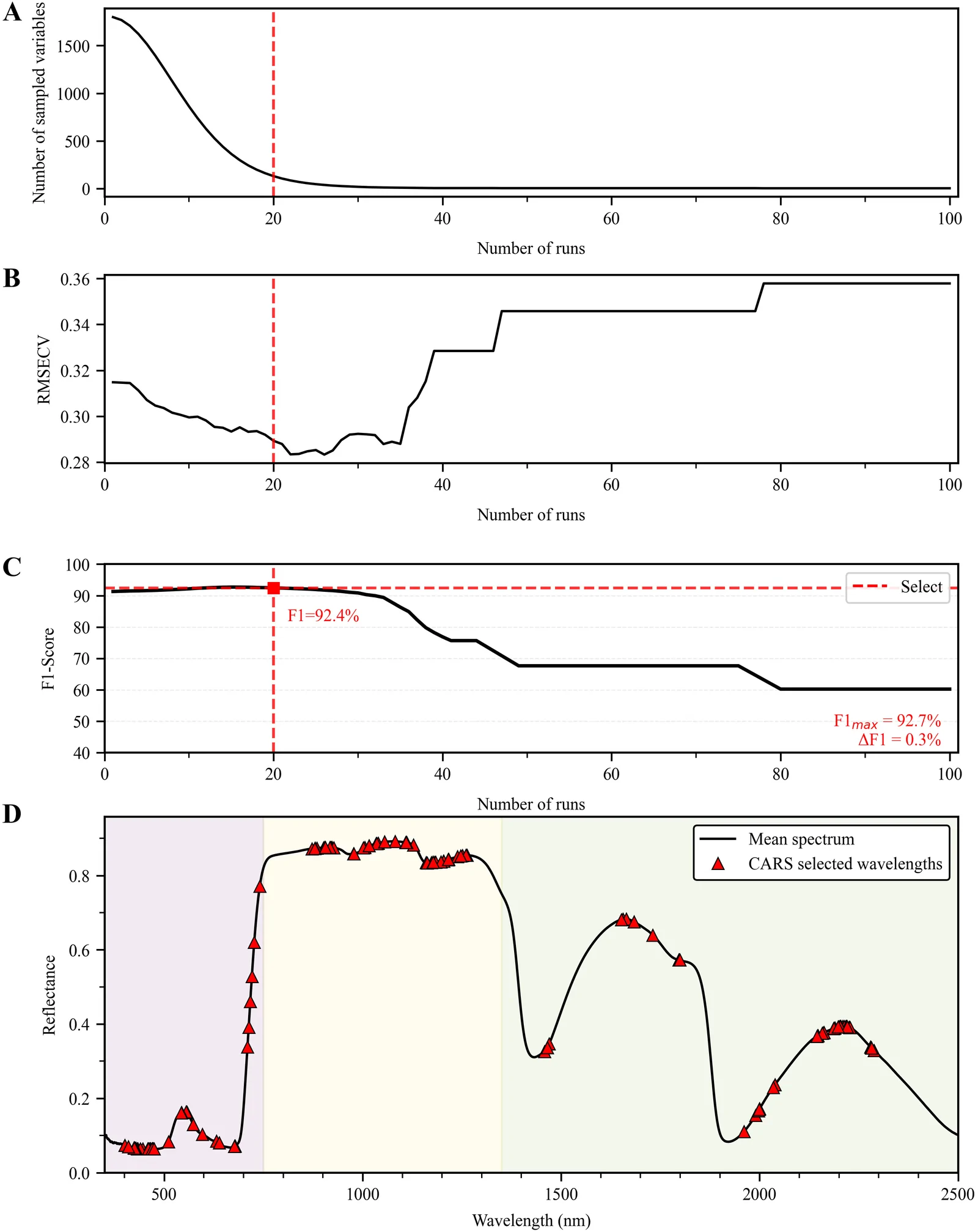
Results of effective wavelengths (EWs) selected by CARS: **(A)** number of sampled variables; **(B)** variation in RMSECV; **(C)** F1-score under the SVM model; **(D)** specific locations of feature bands in the spectrum.

**Figure 7** shows the characteristic wavelength selection process based on SPA. As the number of features increases beyond 44, the decline in RMSECV slows down, and the F1-score starts to level off. Hence, the feature set corresponding to 44 features is optimal (**Figure 7C**).

**Figure 7:**
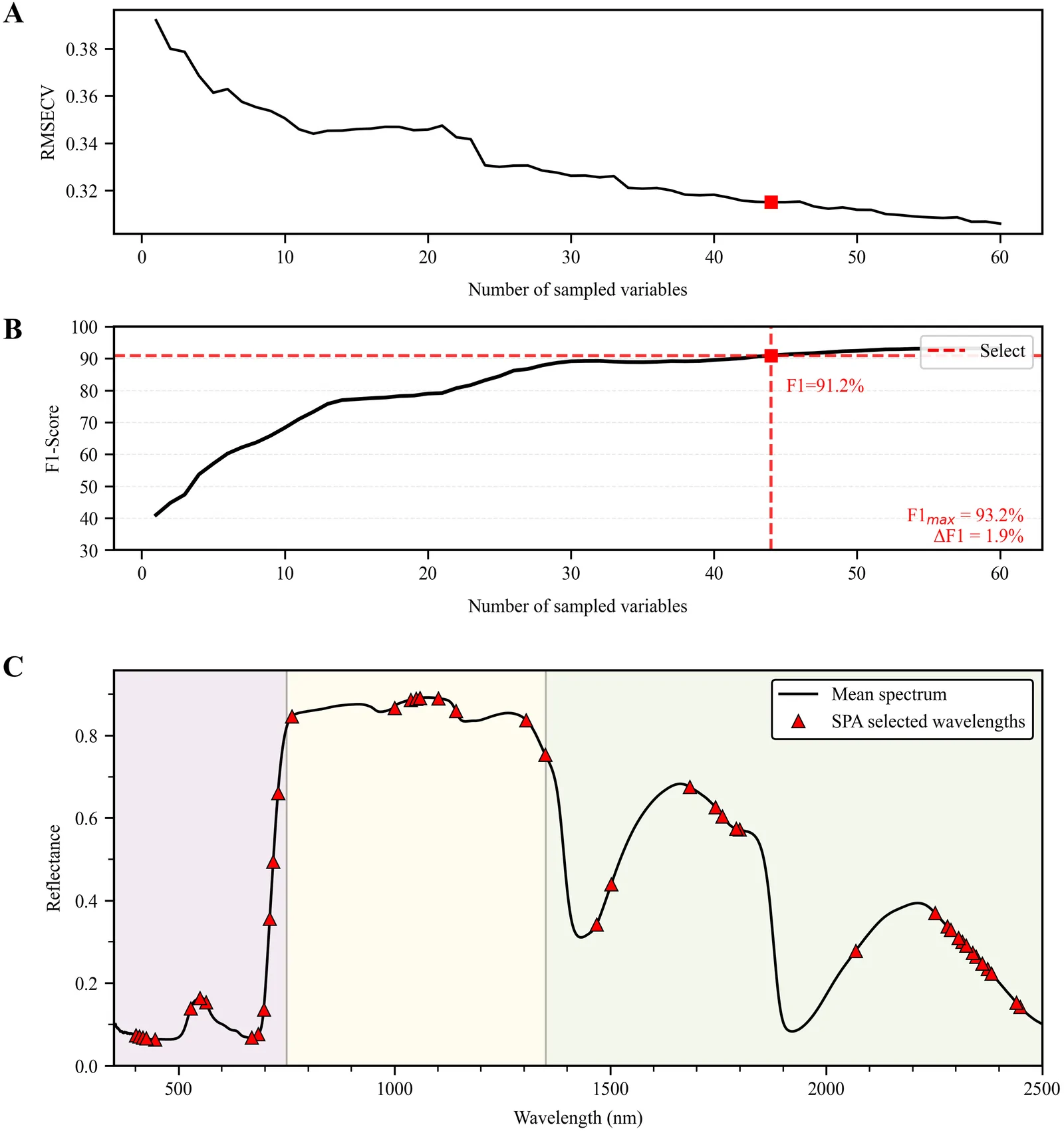
Results of characteristic wavelengths selected by SPA: **(A)** changes in RMSECV; **(B)** F1-score under the SVM model; **(C)** specific locations of characteristic wavelengths in the spectra.

This study attempted to combine the CARS and SPA methods, and the CARS-SPA method retained 19 characteristic wavelengths (**Figure 8**).

**Figure 8:**
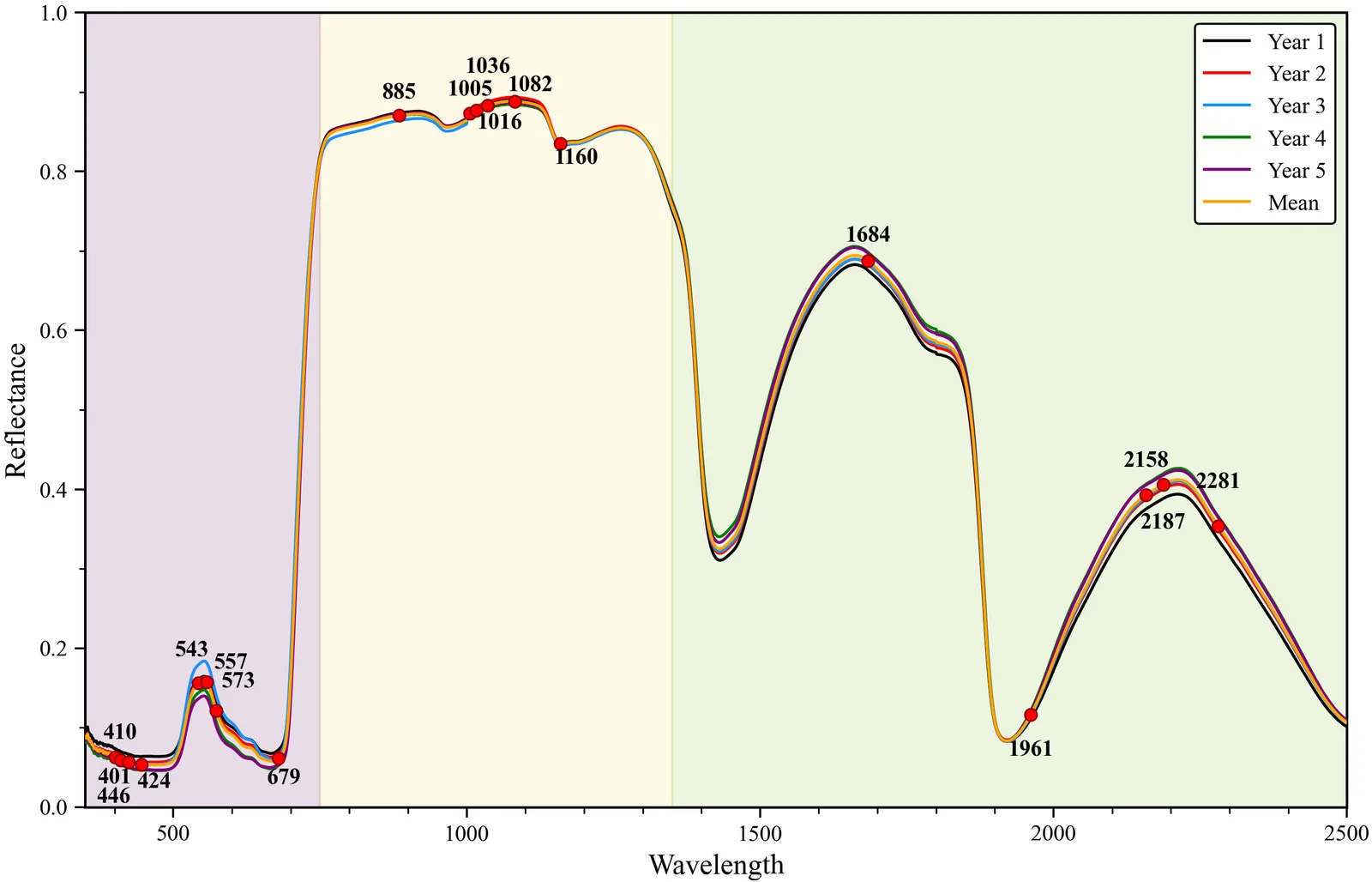
Specific locations of the feature bands selected by CARS-SPA.

#### Modeling analysis

Modeling with feature sets that are based on three different feature extraction methods (**Figure 9**). Of these, the CARS-SPA algorithm retains the fewest characteristic wavelengths, i.e., 19 (0.88% of the full-band data) among all. However, it has high accuracy in all five models with just a slight difference between its accuracy and that of models that were trained on full-band data or feature data selected by CARS.

**Figure 9:**
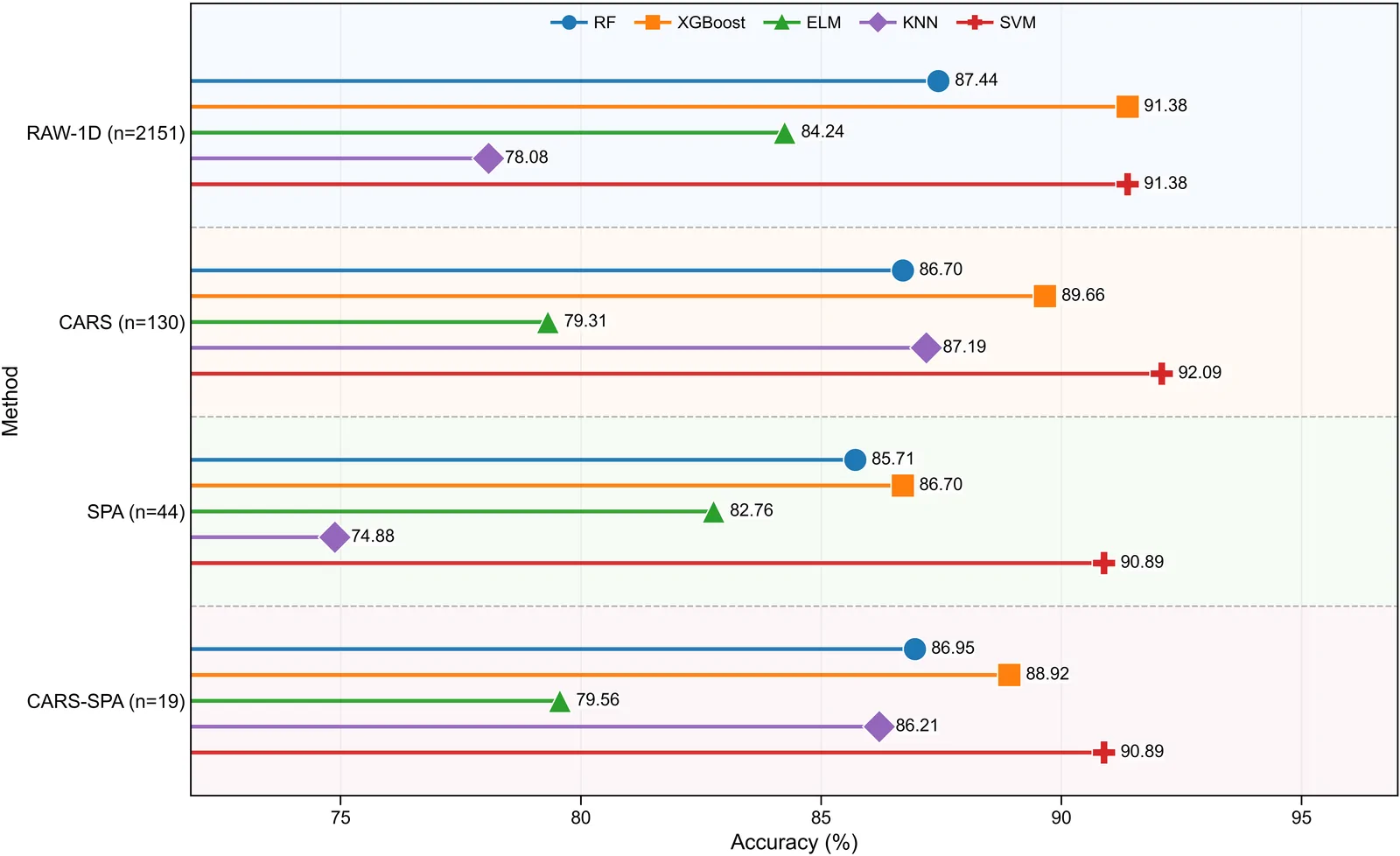
Comparison of classification accuracy using raw data and different feature extraction methods.

Consequently, the 19 characteristic wavelengths selected by CARS-SPA were used as the spectral feature set (Spec) in subsequent experiments and it was found that the SVM model based on that feature set reached 90.89% accuracy.

### Modeling and analysis using fused RGB image data

In this research, modeling was done using spectral features (Spec) and image-derived phenotypic features, with four methods compared: the upper surface of the leaf (ImgA), the lower surface (ImgB), average of upper- and lower-surface features (ImgM), and concatenated upper- and lower-surface features (ImgT) (**Figure 10**).

**Figure 10:**
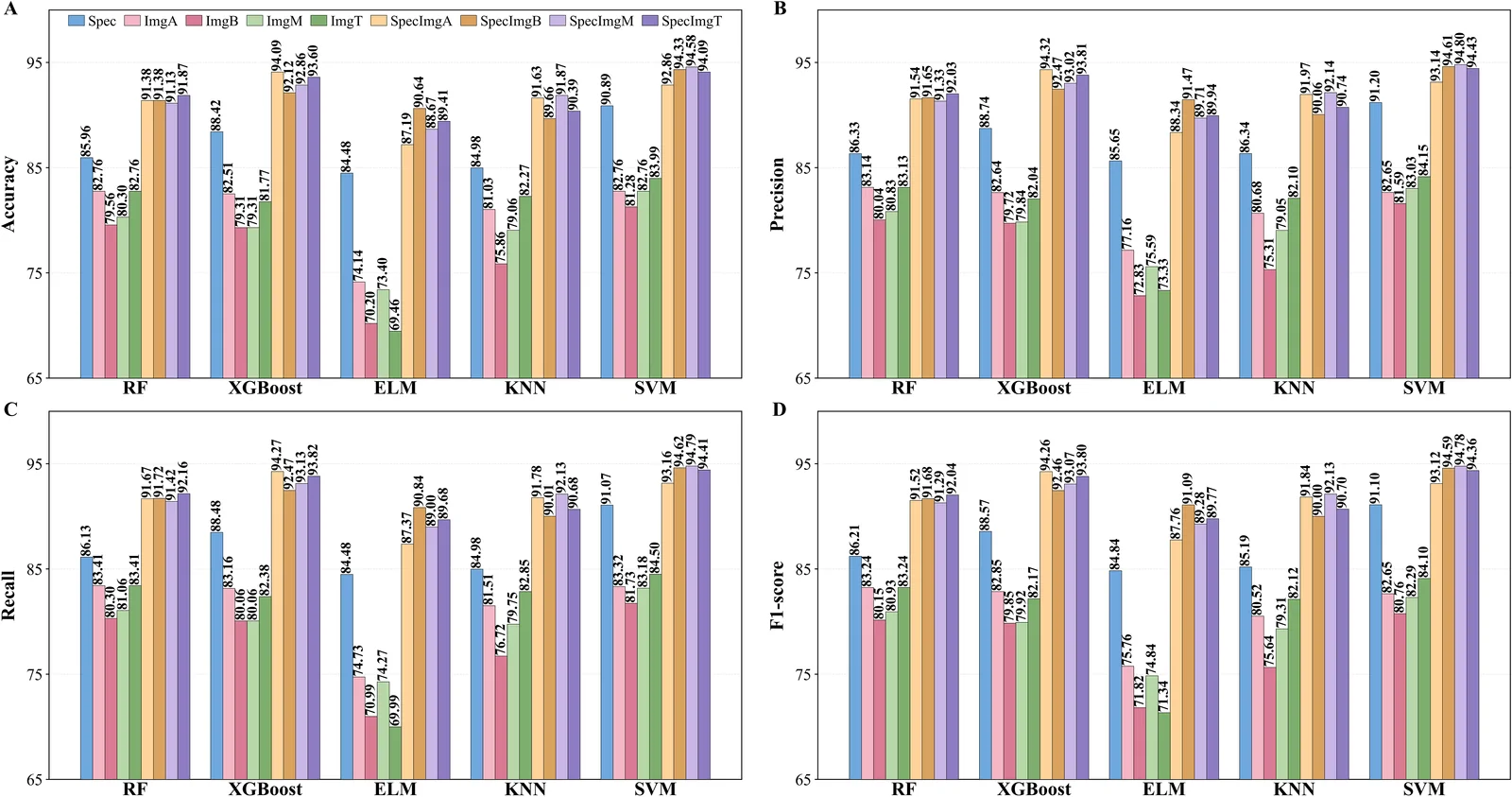
Comparison of the recognition performance of different feature combinations for cultivated ginseng leaves under various classification models (Spec: spectral features; ImgA: upper-surface features; ImgB: lower-surface features; ImgM: average of upper- and lower-surface features; ImgT: concatenated upper- and lower-surface features; SpecImgA: spectral features + upper-surface features; SpecImgB: spectral features + lower-surface features; SpecImgM: spectral features + average of upper- and lower-surface features; SpecImgT: spectral features + concatenated upper- and lower-surface features). **(A)** Accuracy; **(B)** Precision; **(C)** Recall; **(D)** F1-score.

Six features extracted from RGB images (S, R, r_dev, r_skew, Brenner, and Laplacian) are leaf phenotypic features that reflect information about different growth years. The classification accuracies of all models using only RGB image features exceeded 69%. Compared with the lower surface of the leaf, information from the upper surface of the leaf better reflects differences among different growth years, with an accuracy of 82.76% using the RF and SVM models.

Spectral features indirectly characterize physiological and structural differences among leaves based on reflectance information across specific wavelength bands, whereas image features reflect leaf phenotypic information; thus, spectral and image features are fused for modeling (**Figure 10**). Compared to using spectral features alone, the four metrics had relatively large improvements in performance after feature fusion, and Accuracy increased by 1.97 to 6.65 percentage points. The SpecImgM feature set showed relatively stable performance across all models because it combined spectral information with image information from the upper and lower leaf surfaces, having achieved a maximum Accuracy of 94.58% in the SVM model (an improvement of 3.69 percentage points over Spec and 11.82 percentage points over ImgA).

The optimal fusion model was identified by this study as SpecImgM-SVM, and it is used as the basis for subsequent experiments.

To determine whether various combinations of features could be used to obtain information about the changes in growth-year, we did a regression analysis with a support vector regression (SVR) model on nine feature sets (**Figure 11**). In the case of a single feature set, the Spec regression metric performed the worst, and the ImgA metric was the highest (R² = 0.924), and all regression metrics were superior to those of ImgB. All fused feature sets recorded a rise in R² of no less than 0.061 when compared with the Spec feature set, whereas both RMSE and MAE decreased. This also indicates that spectral features and image-derived phenotypic features are complementary, and their fusion could emphasize the differences between different growth years.

**Figure 11:**
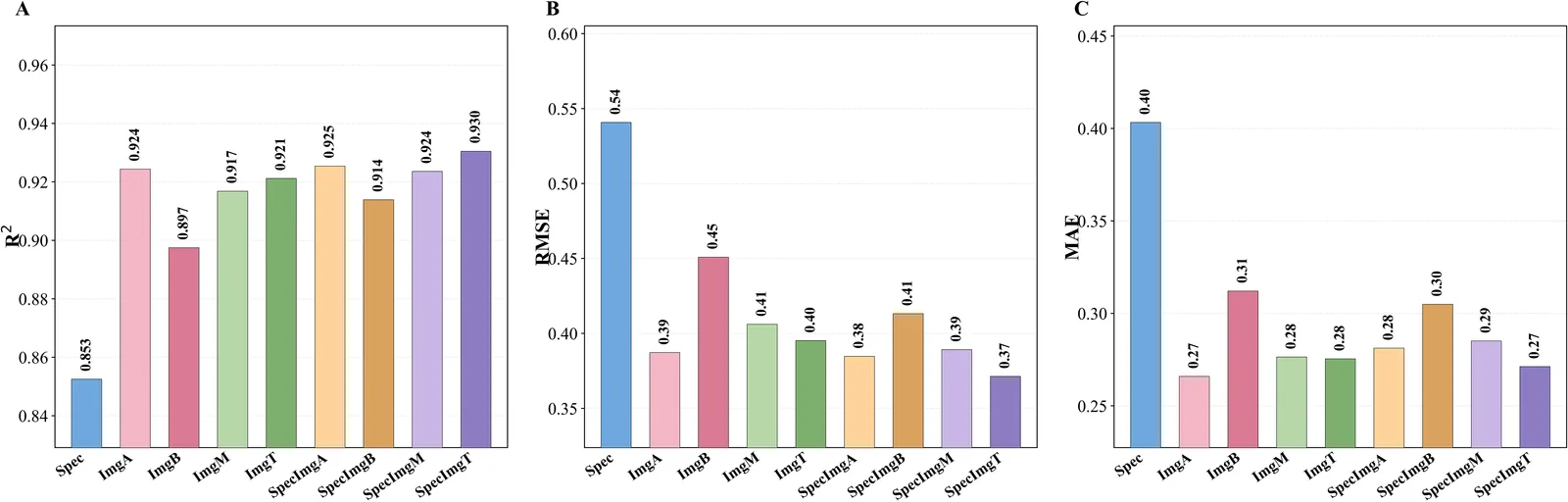
Regression performance of different feature combinations using SVR. **(A)** Coefficient of determination (R^2^); **(B)** Root mean square error (RMSE); **(C)** Mean absolute error (MAE).

### Feature explanation

#### Spectral characteristics

**Table 3** summarizes the physiological significance of the characteristic wavelengths selected by CARS-SPA.

**Table 3:** Distribution of characteristic wavelengths in cultivated ginseng leaves and their physiological significance.

Region	Spectral bands	Physiological significance	References
VIS	401, 410, 424, 446, 543, 557, 573, 679	Mainly affected by absorption by pigments such as chlorophyll and carotenoids.	(Blackburn, 1999)
NIR	885, 1005, 1016, 1036, 1082, 1160	Mainly associated with scattering information from the internal tissue structure of leaves, and may also be affected by weak absorption features of water and some organic groups.	(Jacquemoud and Baret, 1990)
SWIR	1684, 1961, 2158, 2187, 2281	Closely related to absorption features of water and dry-matter components such as lignin, cellulose, and proteins.	(Kokaly and Clark, 1999)

The RF model was applied to rank the characteristic wavelengths according to their importance (**Figure 12**). The four most important characteristic wavelengths were 885 nm, 679 nm, 446 nm, and 401 nm, three of which are within the VIS region.

**Figure 12:**
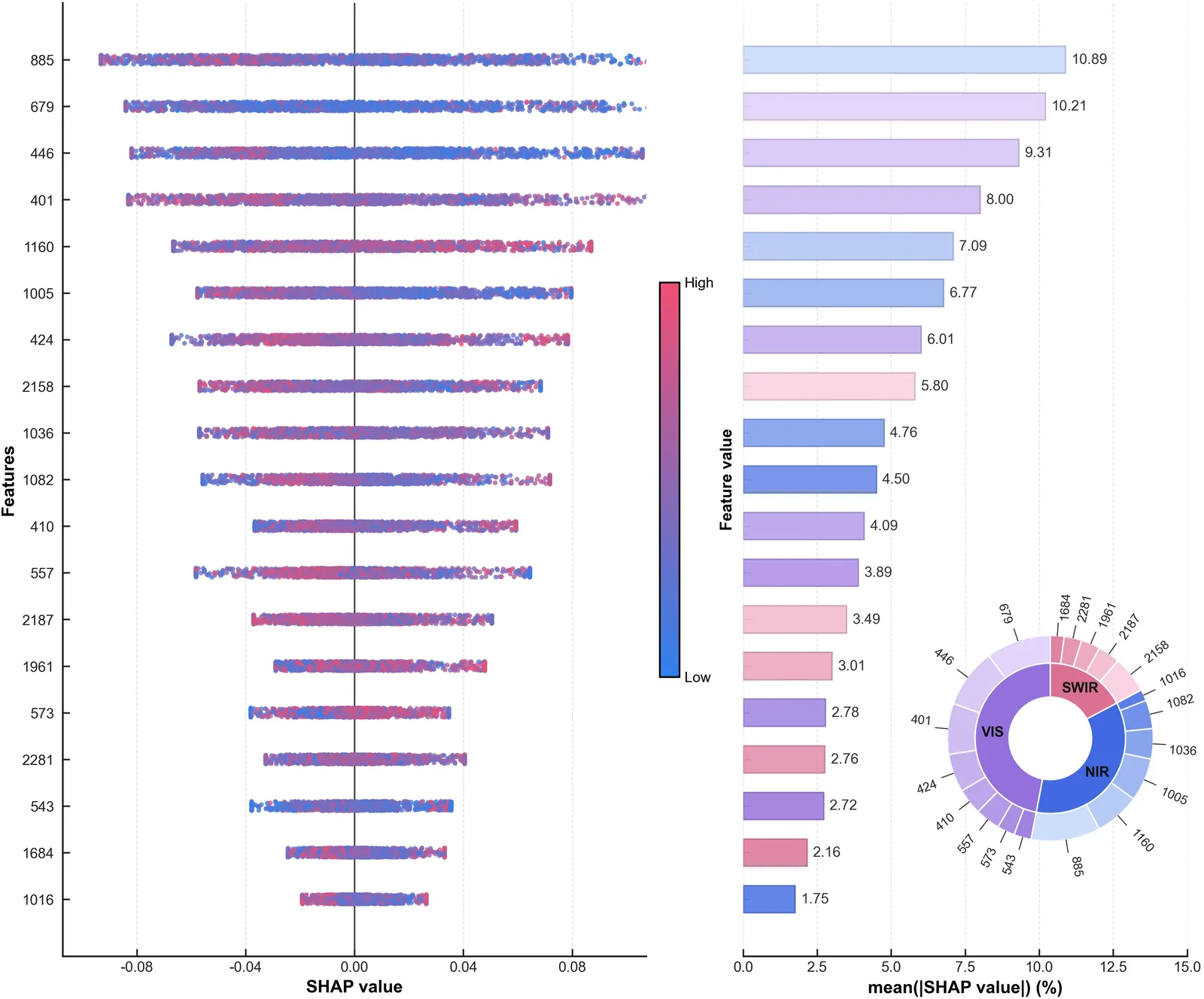
SHAP-based spectral feature importance analysis using RF.

#### Image-derived phenotypic features

**Table 2** summarizes the image-derived phenotypic features and their meanings.

An RF model was used to rank the importance of image-derived phenotypic features (**Figure 13**). The two texture features, Brenner and Laplacian, were significantly more important than other features, and the importance of S was also relatively high.

**Figure 13:**
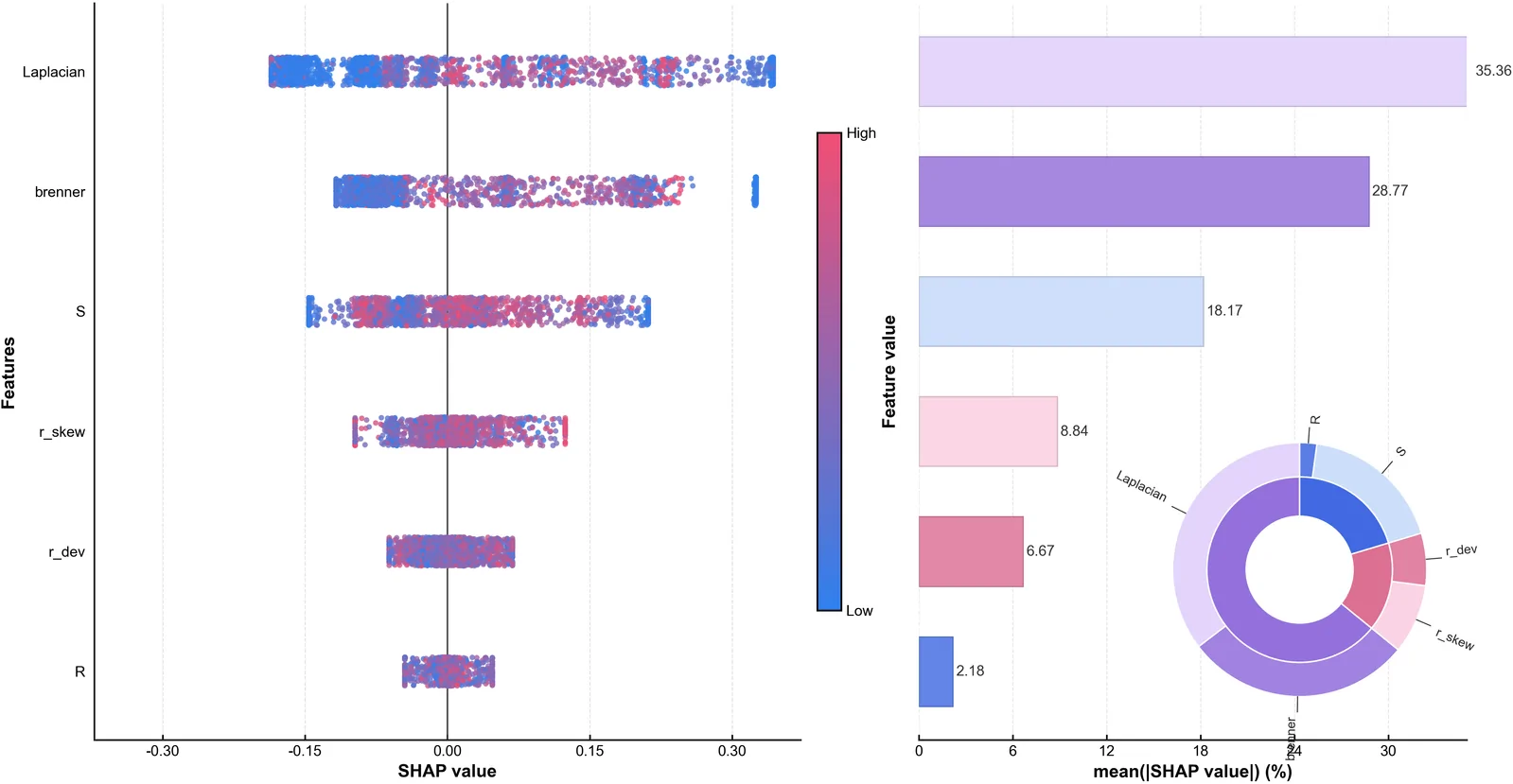
SHAP-based image feature importance analysis using the Random Forest model.

#### Correlation between features and growth years

The Spearman correlation coefficients between different features and different growth years were computed in this research (**Figure 14**). Consider each growth year as a binary variable: assign a value of 1 to the growth year concerned and 0 to all other growth years. 679 nm has a strong correlation with the one-year-old group, the five-year-old group, and the overall 1- to 5-year-old group; 1036 nm and 1082 nm had strong correlations only with the three-year-old group. The correlations between the overall 1- to 5-year-old group and Brenner and Laplacian were particularly strong: 0.934 and 0.895, respectively. A single feature is not sufficient to characterize a particular growth year, as it is only when there are several features working together that they can give information that distinguishes between different growth years.

**Figure 14:**
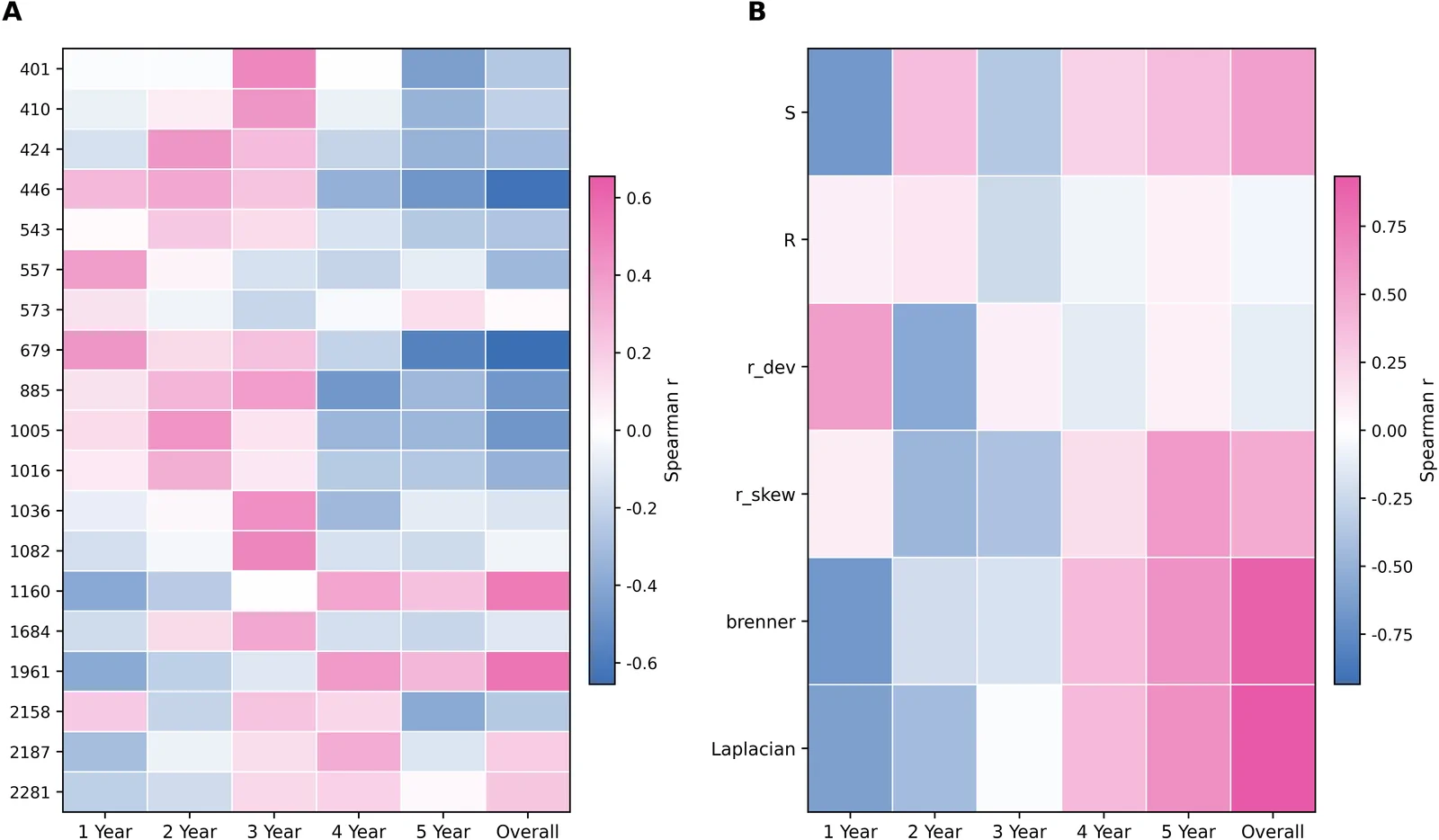
Correlation between features and growth year. **(A)** Correlation between spectral features and growth year; **(B)** Correlation between RGB image-derived phenotypic features and growth year.

### Development and evaluation of a decision-level fusion model combining machine learning and deep learning

SpecImgM-SVM achieved a high overall classification accuracy of 94.58%. In this study, a strategy that combines deep learning and machine learning predictions through voting fusion is employed in order to further enhance the accuracy of predicting the growth year of cultivated ginseng.

Using RGB images of the leaf upper surface (A) and lower surface (B) as inputs, three deep-learning models were used to classify 1- to 5-year-old cultivated ginseng leaves (**Table 4**). The ResNet18 model achieved the highest accuracy of 90.15% with RGB images of the leaf’s lower surface (B) as input. Thus, the ResNet18 model was chosen and RGB images of the leaf’s lower surface were used as input.

**Table 4:** Comparison of deep-learning models.

Model	Group	Accuracy(%)	Precision(%)	Recall(%)	F1(%)
ResNet18	A	89.66	90.15	90.06	90.08
	B	90.15	90.73	90.49	90.53
MobileNetV3	A	60.34	70.61	60.21	60.93
	B	51.74	58.30	56.53	54.25
EfficientNetB0	A	86.21	87.10	86.65	86.82
	B	89.66	90.03	90.10	89.99

The SVM model uses leaf spectral features and RGB image-derived phenotypic features for growth year identification; ResNet18 can be used to automatically extract deeper-level features in images; the two models are based on distinct sources of information. Through decision-level fusion of the predictions from the two models, the SVM-ResNet18 model achieved a five-class accuracy of 96.80% (**Table 5**), which is a 2.22 percentage point improvement over the SVM model (94.58%), as well as a significant reduction in misclassifications between the four- and five-year-old groups (**Figure 15**).

**Table 5:** Evaluation of the classification performance of the SVM-ResNet18 voting fusion model.

Model	Accuracy(%)	95% CI	Precision(%)	Recall(%)	F1(%)
SVM	94.58	92.36 - 96.55	94.80	94.79	94.78
ResNet18	90.15	85.66 – 94.63	90.73	90.49	90.53
SVM-ResNet18	96.80	95.04 - 98.56	96.90	96.94	96.90

**Figure 15:**
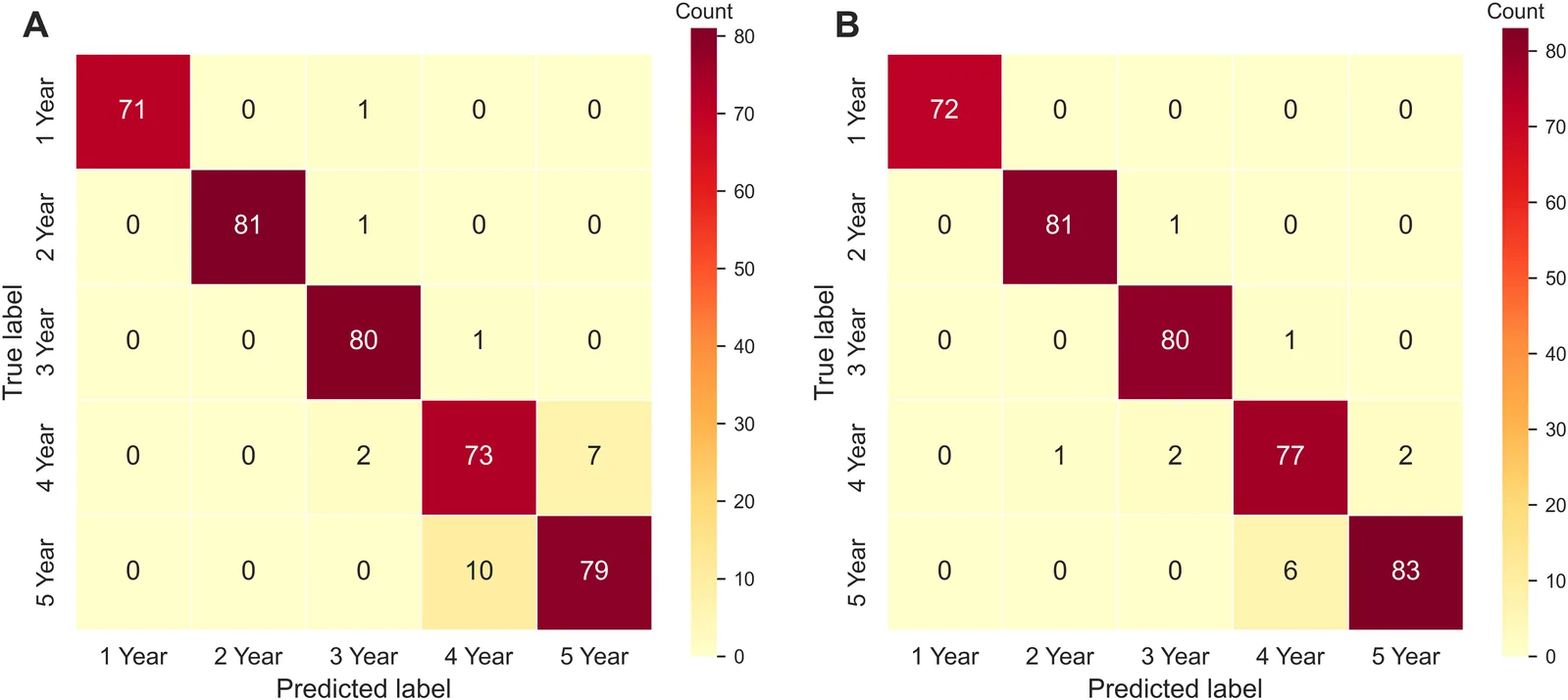
Confusion matrices of different models: **(A)** SVM; **(B)** SVM-ResNet18 voting fusion.

In terms of computational cost, the fusion model required an average training time of 5119.27 s (approximately 1.42 h) during five-fold cross-validation, with an average inference time of approximately 322.37 ms per sample. The hardware specifications used for model training and evaluation are summarized in **Table 6**.

**Table 6:** Hardware specifications used for model training and inference.

Item	Configuration
GPU	NVIDIA GeForce RTX 4060 Laptop GPU
GPU memory	8 GB
CPU	Intel(R) Core(TM) i7-14650HX
RAM	15.78 GB
CUDA version	11.8

## Discussion

The spectral features of the leaves and the RGB image-derived phenotypic features both have a certain degree of separability, and classification performance is enhanced even more upon multimodal fusion. This indicates that both the spectral and phenotypic information of leaves contain growth-year-sensitive features, and thus the use of leaves as samples for growth-year identification tasks is feasible.

Hyperspectral data may indirectly indicate the differences in pigments, water content, and tissue structure within the leaves through the measurement of reflectance information from cultivated ginseng leaves across continuous bands. With an increase in the growth year, it could be possible that the degree of leaf development as well as the condition of its internal tissues change. Thus, the spectral data of cultivated ginseng leaves from different growth years has a certain degree of separability. This study indicates that separability of cultivated ginseng from different growth years is not attributable to changes in a single trait. Various spectral bands offer information about leaf condition from different perspectives and together, several spectral bands can all reveal differences between leaves of different growth years.

The shape, color, and texture of cultivated ginseng leaves can be modified due to changing of growth years and RGB images are very suitable in depicting such changes. The morphological features S and R indicate the size and shape of the leaf, whereas the color features r_dev and r_skew represent the dispersion and asymmetry of the red component respectively, and the features Brenner and Laplacian indicate the variation in leaf edge details and the richness of texture. If it comes to these six phenotypic features, the RGB images of the leaf’s upper surface are more indicative of the variations across different growth years.

The spectral features describe differences in the internal physiological structure of leaves across different growth years, whereas the RGB image-derived phenotypic features reflect differences in the external phenotype of the leaves. Because the two information sources are different, their fusion provides complementary information, thereby significantly improving the ability to classify cultivated ginseng leaves from different growth years.

RGB image-derived phenotypic features have limited expressive power, so a deep-learning-based five-class classification model was developed to make full use of the information contained in RGB images. The machine-learning model can use the selected spectral features and RGB image-derived phenotypic features to effectively identify different growth years from the physiological and phenotypic perspectives of leaves, whereas the deep-learning model is able to acquire more complex visual features using RGB images. The combination of the predictions from the machine-learning feature fusion models and those from the deep-learning models by means of decision fusion can enhance the stability and recognition accuracy of the models.

The given research paper also has a number of drawbacks. To begin with, all samples came from one cultivation spot and were obtained on the same day. Thus, the existing model quality is largely determined by the dissimilarity of the multimodal features of ginseng leaves of different growth years grown under the same experimental conditions. The sampling approach was useful to minimize the influence of environmental variations at the time of the data collection enabling the model to better access the information related to the spectral and phenotypic features of the growth years. Nevertheless, there might be some restrictions on the generalization potential of the model in case of multifaceted production conditions due to the use of one cultivation site and one acquisition time. The difference in climatic conditions, soil attributes, illumination conditions, and cultivation management techniques in various cultivation areas can change the physiological state, pigment composition, tissue structure, and outer morphology of ginseng leaves, which can subsequently impact the spectral responses and the RGB image-derived phenotypic features. Hence, it is not possible to directly presuppose the applicability of the model to other regions of cultivation or growth conditions but should be evaluated across regions and environments.

However, the suggested multimodal fusion model combines with the ability of hyperspectral data to identify inner physiological changes of leaves with the power of RGB images to represent outer phenotypic features to offer a viable solution to non-destructive identification of ginseng growth years. As it is based largely on extensive phenotypic features that are obtained through leaves as opposed to environmental data pertinent to one location, it can still be used in other environmental study areas in the future. The future studies ought to develop more heterogeneous datasets including various geographical locations, time of acquisition and growing conditions and use independent external validation measures to determine the transferability, robustness, and practical applicability of models to enhance the use of this technique in practical ginseng production.

## Conclusions

Using 1- to 5-year-old cultivated ginseng leaves as samples, the presented research developed a machine-learning and deep-learning dual-branch fusion model for non-destructive identification of the growth year of cultivated ginseng. The accuracy of the SVM model based on raw spectral data of cultivated ginseng leaves was 59.36%. After applying a 1D transformation and performing feature selection using CARS-SPA, 19 characteristic wavelengths were retained (accounting for only 0.88% of the full spectrum), and the accuracy improved to 90.89%. By performing feature fusion on 19 spectral features and 6 RGB image-derived phenotypic features, the accuracy was further improved to 94.58%. Through decision fusion between the SVM and ResNet18 models, the accuracy was improved by 2.22 percentage points to 96.80%. It is shown that the two-stage fusion strategy proposed in this study is effective in improving the accuracy of identifying the growth year of cultivated ginseng and enables rapid, non-destructive determination of the growth year. At the same time, this method also serves as a reference for identifying the growth stages or varieties of other crops.

## Data Availability

The raw data supporting the conclusions of this article will be made available by the authors, without undue reservation.
